# Metabolomics hallmarks *OPA1* variants correlating with their *in vitro* phenotype and predicting clinical severity

**DOI:** 10.1093/hmg/ddaa047

**Published:** 2020-03-23

**Authors:** Juan Manuel Chao de la Barca, Mario Fogazza, Michela Rugolo, Stéphanie Chupin, Valentina Del Dotto, Anna Maria Ghelli, Valerio Carelli, Gilles Simard, Vincent Procaccio, Dominique Bonneau, Guy Lenaers, Pascal Reynier, Claudia Zanna

**Affiliations:** 1 Département de Biochimie et Génétique, Centre Hospitalier Universitaire, 49933 Angers, France; 2 Equipe Mitolab, Institut MITOVASC, CNRS 6015, INSERM U1083, Université d'Angers, 49035 Angers, France; 3 Department of Pharmacy and Biotechnology (FABIT), University of Bologna, 40126 Bologna, Italy; 4 Unit of Neurology, Department of Biomedical and NeuroMotor Sciences (DIBINEM), University of Bologna, 40126 Bologna, Italy; 5 IRCCS Istituto delle Scienze Neurologiche di Bologna, UOC Clinica Neurologica, 40139 Bologna, Italy

## Abstract

Interpretation of variants of uncertain significance is an actual major challenge. We addressed this question on a set of *OPA1* missense variants responsible for variable severity of neurological impairments. We used targeted metabolomics to explore the different signatures of *OPA1* variants expressed in *Opa1* deleted mouse embryonic fibroblasts (*Opa1*^−/−^ MEFs), grown under selective conditions. Multivariate analyses of data discriminated *Opa1^+/+^* from *Opa1^−/−^* MEFs metabolic signatures and classified *OPA1* variants according to their *in vitro* severity. Indeed, the mild p.I382M hypomorphic variant was segregating close to the wild-type allele, while the most severe p.R445H variant was close to *Opa1^−/−^* MEFs, and the p.D603H and p.G439V alleles, responsible for isolated and syndromic presentations, respectively, were intermediary between the p.I382M and the p.R445H variants. The most discriminant metabolic features were hydroxyproline, the spermine/spermidine ratio, amino acid pool and several phospholipids, emphasizing proteostasis, endoplasmic reticulum (ER) stress and phospholipid remodeling as the main mechanisms ranking *OPA1* allele impacts on metabolism. These results demonstrate the high resolving power of metabolomics in hierarchizing *OPA1* missense mutations by their *in vitro* severity, fitting clinical expressivity. This suggests that our methodological approach can be used to discriminate the pathological significance of variants in genes responsible for other rare metabolic diseases and may be instrumental to select possible compounds eligible for supplementation treatment.

## Introduction

High-throughput sequencing currently reports myriads of genetic variants for which pathogenicity is difficult to predict in the absence of universal functional tests. Metabolomics, which is closer to the phenotype than any other ‘omics,’ may be particularly relevant for such fine pathogenicity prediction.

More than 400 pathogenic variants have been reported in *OPA1* (Optic Atrophy 1) gene (http://opa1.mitodyn.org) (1, 2), responsible for optic nerve degeneration and visual loss, ranging from isolated ‘Dominant Optic Atrophy’ (DOA; OMIM #165500) to more severe multi-systemic syndromes named ‘DOA*plus*’ (OMIM #125250), including some bi-allelic cases with Behr Syndrome (OMIM #210000). *OPA1* encodes a dynamin-related GTPase driving the efficiency of energy production through its multiple roles in mitochondrial dynamics and ultrastructure (3–6), mitochondrial DNA (mtDNA) maintenance (7–9), apoptosis (10), calcium fluxes (11), oxidative stress (12), mitophagic flux (13–15), and more generally in mitochondrial plasticity and quality control (16–18), all these functions being affected when *OPA1* is mutated.

The severe DOA*plus* syndromes, affecting about 20% of patients, involve extraocular features such as sensorineural deafness, ataxia, peripheral neuropathy and chronic progressive external ophthalmoplegia with mitochondrial myopathy and mtDNA instability (19). Additional neurological phenotypes, such as spastic paraplegia (19), multiple sclerosis-like syndrome (20), parkinsonism and dementia (21), have also been related to dominant *OPA1* variants. More recently, the severe early onset Behr (22–24), MELAS-like stroke (25) and Leigh-like (26) syndromes, as well as syndromes involving cardiomyopathy (27), were associated with biallelic *OPA1* inheritance (for reviews see 17, 28).

The relationships between *OPA1* variants and their clinical consequences remain largely unknown. Only two clear cut genotype/phenotype correlations have been established so far: one concerns the higher severity of missense mutations in the GTPase and dynamin domains than those leading to OPA1 haplo-insufficiency (19). For instance, the p.R445H variant has been associated with severe dominant forms of the disease (29). The second is related to *OPA1* bi-allelic inheritance leading to severe encephalopathy with, sometimes, early death of the patients. Although a few cases of homozygous variants were reported, the majority of clinical presentations associated with bi-allelic inheritance are related to the association of an *OPA1* pathogenic variants with a rather frequent p.I382M hypomorphic *OPA1* variant, which by itself, either in heterozygous or homozygous state, has almost no clinical consequence (22–24, 30).

To better understand the impact of *OPA1* pathogenic variants on mitochondrial functions, we cloned mutated cDNAs of the human *OPA1* isoform 1 (ISO1), and stably expressed them in *Opa1*^−/−^ MEFs (31). These isogenic cell models revealed that the p.D603H variant, associated with isolated optic atrophy, exhibited mildly reduced respiration and complex V disassembly with increased mitochondrial network fragmentation. The most severe p.G439V and p.R445H variants, associated with DOA*plus* displayed a complete mitochondrial network fragmentation, severe energetic impairment and mtDNA depletion. Conversely, the hypomorphic p.I382M variant alone failed to manifest any significant mitochondrial alteration, under normal growth condition in high-glucose medium (31).

Here, we used a targeted metabolomics approach to re-examine this collection of isogenic cells bearing *OPA1* wild-type and pathogenic variants in glucose-free medium supplemented with galactose, a growth condition forcing the oxidative metabolism (32). We evidenced a clear correlation between the metabolomic signature of each mutation, including the hypomorphic p.I382M variant, with their *in vitro* phenotype, overall fitting the clinical severity, thus predicting with high resolution their pathogenicity and clearly discriminating among the different missense variants.

## Results

### Metabolomics analysis

**Figure 1:**
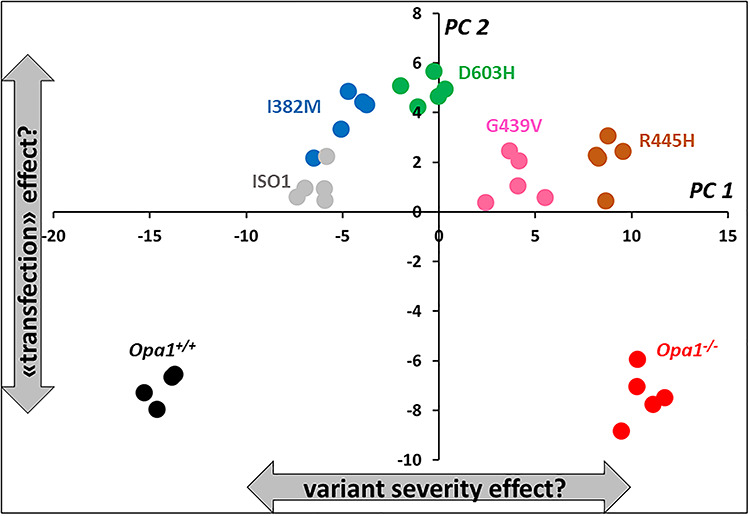
First principal plan of the PCA. When samples are projected into the first principal component (PC1) different isoforms are well separated according to their proximity to *Opa1^+/+^* (black circles) or *Opa1*^−/−^ (red circles) which have opposite coordinates in PC1 and represent normal and totally deleted *Opa1* gene, respectively. Two subgroups are less well separated though: transfected human normal isoform (ISO1, gray circles) from I382M (blue circles) variant and R445H (brown circles) variant from totally deleted murine *Opa1^−/−^* MEF. When projecting samples into the second PC (PC2) non-transfected MEF (*Opa1*^+/+^ and *Opa1*^−/−^) cannot be separated from each other but plot far away from the projection of transfected MEFs in PC2, indicating a possible effect of transfection. Samples transfected with D603H and G439V isoforms have been represented as green and pink circles, respectively.

We used a targeted metabolomics approach on *Opa1^+/+^*, *Opa1^−/−^* MEFs and five *Opa1^−/−^* MEFs expressing human WT or mutated ISO1 OPA1, grown for 24 h in galactose medium. After validation of the quality control (QC) and considering the dynamic range for each metabolite, 129 out of 188 (68.6%) metabolites were conserved for statistical analyses (Supplementary Table S1). First plan of the principal component analysis (PCA) showed that sample normalization was accurate to overcoming batch effect (Supplementary Fig. S1). The first principal plan of the PCA with all seven groups of MEFs showed a clear discrimination between groups with the first and the second principal components (PC1 and PC2), accounting for more than 68% of the total data covariance (Fig. 1). PC1 accounts for 51.3% of the total covariance and when samples are projected to this PC groups are distinctively separated according to the function of OPA1 gene and the impact different isoforms have on the clinical presentation of the disease based on genotype-phenotype correlation. PC2 captures 17.1% of the total covariance and when the samples are projected into this component, MEFs transfected with human OPA1 has positive coordinates (positive score values) whilst MEFs with native murine *Opa1* gene have negative score values suggesting an effect of the transfection experience on MEF metabolic phenotype. Independence of these two factors (impact of gene mutation and transfection) on the metabolome is verified here by the orthogonality of PCs.

**Figure 2:**
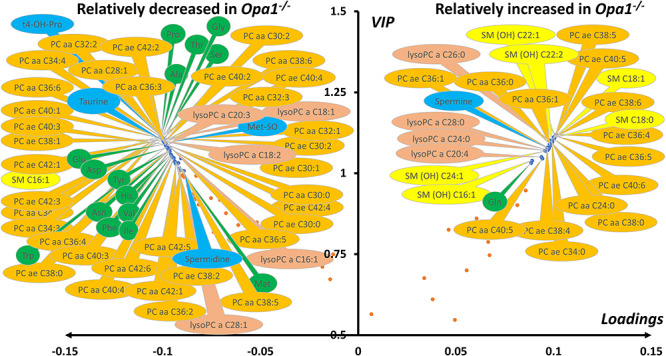
Volcano plot (loadings versus VIP) from the OPLS-DA model comparing murine *Opa1^+/+^* and *Opa1^−/−^* MEF. Only most important metabolites, i.e. those with VIP ≥ 1, have been labelled. Negative loadings indicate relatively decreased metabolite concentrations in *Opa1^−/−^* compared to *Opa1^+/+^* MEF. Main features of the metabolomic signature are decreased level in *Opa1^−/−^* MEF of many dialkyl and acyl-alkyl (PC aa and PC ae) phosphatidylcholine species (orange bubbles), almost all amino acids (green bubbles), some biogenic amines (blue bubbles) including spermidine, trans-hydroxyproline (t4-OH-Pro), taurine and methionine-sulfoxide (Met-SO) along with lysophosphatidylcholines (lysoPC, brown bubbles) with acyl chain length of less than 22 carbons. On the other side, many sphingomyelins (SM, yellow bubbles), mainly hydroxylated (SM(OH)), some phosphatidylcholine species, the highest order polyamine spermine and lysophosphatidylcholines species with acyl chain with more than 22 carbons were found increased in *Opa1^−/−^* compared to *Opa1^+/+^* MEF. Glutamine was the only amino acid relatively increased in *Opa1^−/−^* MEF. Legend: Ala, alanine; Asn, asparagine; Asp, aspartate; Gln, glutamine; Gly, glycine; His, histidine; Ile, isoleucine; Met, methionine; Phe, phenylalanine; Pro, proline; Ser, serine; Thr, threonine; Trp, tryptophan; Val, valine. For phosphatidylcholines, the sum of the length of the two acyl or acyl–alkyl groups is noted after the C and is followed by the number of double bonds. The same notation is used for representing the length and the number of double bonds in the acyl chain of sphingomyelins and lysophosphatidylcholines.

As expected from the PCA, the supervised orthogonal projections to latent structures discriminant analysis (OPLS-DA) (not shown) model separated *Opa1^+/+^* and *Opa1^−/−^* samples with excellent predictive capabilities and very low risk of overfitting (*Q^2^Y*_cum_ = 0.99; *Q^2^Y*_cum*-*perm_ = −0.82 and *P*-value _CV-ANOVA_ = 1.35e-5). Important metabolites contributing to this model according to their variable importance in projection (VIP) and loadings are represented in Figure 2. Metabolic signature was essentially composed by relatively diminished concentration in MEF bearing deleted *Opa1* gene of 15 out of 20 quantified amino acids, the indicator of collagen turnover trans-4-hydroxyproline and 3 other biogenic amines: taurine, methionine sulfoxide (Met-SO) and the polyamine spermidine. Glutamine was the only amino acid found relatively increased in *Opa1^−/−^* MEF fibroblasts while the polyamine spermine was found also relatively increased in these cells. Concerning lipid molecules, there exists a deep phosphatidylcholine (PC) remodeling with rather decreased PCs concentration in *Opa1^−/−^* MEF whilst the opposite is observed with sphingomyelins (SM), except for SM 16:1. Lysophosphatidylcholines (lysoPC) even when participating to the metabolic signature did not behave as a single class and some lysoPC were found relatively increased while other relatively decreased in *Opa1^−/−^* MEF.

**Figure 3:**
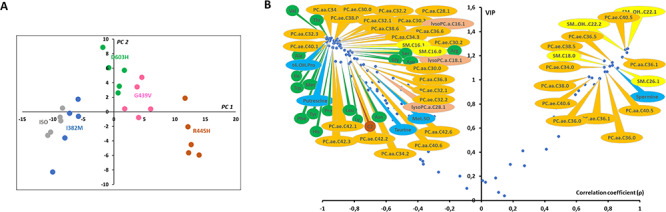
Scatter plots of the PCA first principal plan (**A**) and the Spearman correlation coefficient (ρ) versus VIP (**B**) comparing MEF metabolomes transfected with five human *OPA1* variants. (A) Out of 5, 4 groups are easily distinguishable in the first principal component (PC1): ISO1- I382M, D603H, G439B and R445H. No group discrimination can be seen in the second principal component (PC2). (B) Spearman correlation coefficients (ρ) where obtained from the non-parametric correlation between sample’s scores in the first principal component or t1 in (A) and each metabolite concentration whilst VIP comes from the OPLS model. Only most important metabolites, i.e. those with VIP ≥ 1, have been labelled. For a given metabolite, negative values of ρ indicate negative correlation between its concentration and t1 (i.e. metabolite concentration are relatively close to *Opa1*^+/+^ MEF concentration and relative far from *Opa1*^−/−^ MEF concentration) whilst a positive ρ value indicates the opposite situation (i.e. metabolite concentration are relatively close to *Opa1*^−/−^ MEF concentration and relative far from *Opa1*^+/+^ MEF concentration. Main features of the metabolomic signature are decreased level of many dialkyl and acyl-alkyl (*PC aa* and *PC ae*) phosphatidylcholine species (orange bubbles), almost all amino acids (green bubbles), some biogenic amines (blue bubbles) including taurine, putrescine, trans-4-hydroxyproline (*t4-OH-Pro*) and methionine-sulfoxide (*Met-SO*) along with three lysophosphatidylcholines (*lysoPC*, brown bubbles) and two sphingomyelins (*SM*, yellow bubbles). On the other side, four sphingomyelins (*SM*, yellow bubbles), two of them hydroxylated (*SM(OH)*), some phosphatidylcholine species and the highest order polyamine spermine were found to increase while approaching the metabolomic signature of *Opa1*^−/−^. Legend: Ala, alanine; Arg, arginine; Asn, asparagine; Asp, aspartate; Gly, glycine; His, histidine; Ile, isoleucine; Leu, leucine; Lys, lysine; Met, methionine; Phe, phenylalanine; Pro, proline; Ser, serine; Thr, threonine; Trp, tryptophan; Ser, serine; Val, valine. For phosphatidylcholines, the sum of the length of the two acyl or acyl–alkyl groups is noted after the C and is followed by the number of double bonds. The same notation is used for representing the length and the number of double bonds in the acyl chain of sphingomyelins and lysophosphatidylcholines.

When only *Opa1^−/−^* MEFs with transfected human *OPA1* isoforms were studied, the first two PC explained 65.5% of the total covariance in the X matrix (containing only metabolomic data from transfected isoforms), with PC1 and PC2 accounting for 52.5 and 13%, respectively (Figure 3A). Out of 5, 4 groups formed by OPA1 isoforms were unambiguously distinguished in the first component of the PCA. Indeed, only ISO1 (wild-type allele) and p.I382M isoforms were not clearly separated one another in this component. To tackle the relation depicted by the PCA, an unsupervised method that ignores any response vector, we decided to build a supervised OPLS model considering coordinates in PC1 (called scores or t1 values) as the quantitative Y vector. We also calculate Spearman correlation coefficient between t1 and each metabolite in the X matrix. As expected from the PCA, the predictive latent variable (pLV) of the OPLS model correlated very well with the expected Y vector (not shown). This model had excellent predictive capabilities and the risk of overfitting was very low (*Q^2^Y*_cum_ = 0.99.; *Q^2^Y*_cum-perm_ = −0.34 and *P*-value _CV-ANOVA_ = 2.9e−28). Important metabolites for the explanation of Y vector (i.e. t1 scores as indicators of group separation) are represented as a volcano plot combining univariate and multivariate analysis with Spearman ρ correlation coefficient in the x-axis and VIPs of the OPLS model in the y-axis (Figure 3B). Concentration of 17 out of 20 amino acid correctly measured were higher in the fibroblasts carrying OPA1 isoforms associated to more severe clinical phenotype (p.G439V and p.R445H). When the metabolic phenotype was oriented toward *Opa*1^−/−^ metabolic phenotype, concentrations of trans-4-hydroxyproline, taurine, methionine sulfoxide and the low protonated polyamine putrescine decreased whilst the concentration of the highest protonated spermine increased. As the metabolome get closer to the *Opa1*^−/−^ metabolic phenotype concentration of 23 PCs, 2 SMs, 3 lysoPCs and acetyl-carnitine (C2) decreased whilst the concentration of 11 PCs and 4 SMs increased.

**Figure 4:**
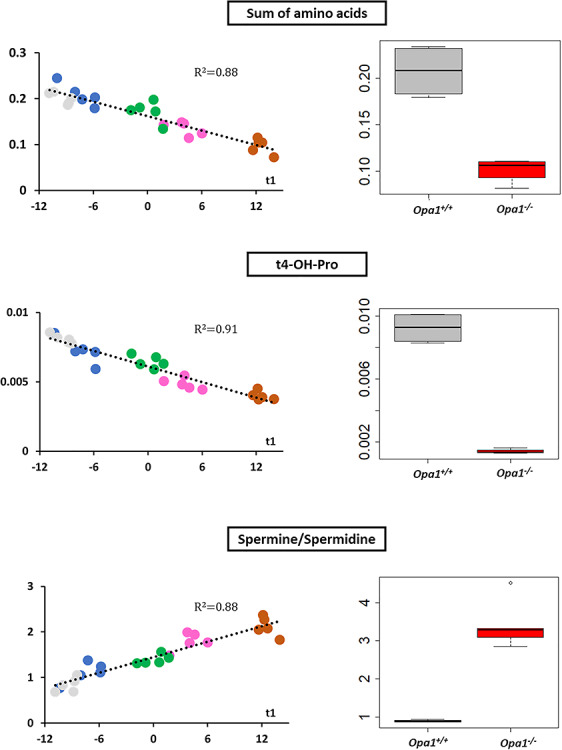
Regression and box plots for polar metabolites. Relative metabolite concentrations for the sum of amino acids (upper panel), trans-4-hydroxyproline (t4-OH-Pro) (middle panel) and the ratio spermine to spermidine (downer panel) have been linearly regressed with t1 (scores of PC1 of the PCA for transfected cells) (left*)* or represented as a box plots for *Opa1*^+/+^ and *Opa1*^−/−^ MEFs (right). In MEFs transfected with human isoforms of *OPA1* gene, regression lines have been drawn along with their respective determination coefficients (*R*^2^). Amino acid and t4-OH-Pro linearly decrease whilst spermine synthase activity, measured by spermine/spermidine ratio, linearly decreases with t1 (i.e. when metabolic phenotype approaches *Opa1*^−/−^ and get far away from *Opa1*^−/−^ phenotypes). These changes are obviously verified in *Opa1*^+/+^ and *Opa1*^−/−^ MEFs. In regression plots, the following color code has been used: ISO1 (gray); I382M (blue); D603H (green); G439V (pink) and R445H (brown). In box plots *Opa1*^+/+^ (WT) box has been colored in dark gray whilst red was used for *Opa1*^−/−^ boxes.

**Figure 5:**
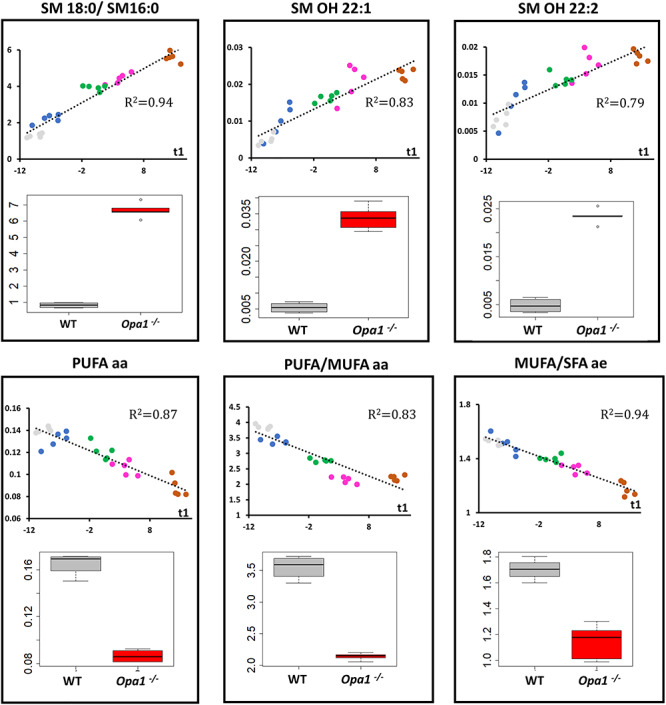
Regression and box plots for lipid metabolites*.* Upper panels: relative metabolite concentrations for hydroxy sphingomyelins SM OH 22:1 and SM OH 22:2 (upper middle and right panels, respectively) and the ratio between octadecanoyl (SM 18:0) and hexadecanoyl (SM 16:0) sphingomyelins (upper left panel) have been linearly regressed with t1 (scores of PC1 of the PCA for transfected cells) or represented as box plots for *Opa1*^+/+^ and *Opa1*^−/−^ MEFs (bottom position in each panel). As for polar metabolites, in MEFs transfected with human isoforms of *OPA1* gene, regression lines have been drawn along with their respective determination coefficients (*R*^2^). There exists a clear parallel increasing between the two structurally close related SM OH 22:1 and SM OH 22:2 and t1. Also, the proportion of SM 18:0 seems to increase when the metabolic phenotype approaches that of *Opa1*^−/−^ compared to the concentration of SM 16:0. Bottom panels: the sum of polyunsaturated diacyl phosphatidylcholines (PUFA aa) (bottom left) and the ratios between PUFA aa and monounsaturated diacyl phosphatidylcholines (PUFA/MUFA aa) (bottom middle*)* and between monounsaturated and saturated fatty acids in the alkyl-acyl family (MUFA/SFA ae) (bottom right*)* decrease linearly with t1. As expected these changes are verified in *Opa1*^+/+^ (WT) and *Opa1*^−/−^ MEFs. In regression plots, the following color code has been used: ISO1 (gray); I382M (blue); D603H (green); G439V (pink) and R445H (brown). In box plots *Opa1*^+/+^ (WT) box has been colored in dark gray whilst red was used for *Opa1*^−/−^ boxes.

OPLS model shared 44 (67%) important metabolites with the OPLS-DA model discriminating *Opa1*^+/+^ from *Opa1*^−/−^ −carrying MEFs. All metabolic signatures of *OPA1* variants shared a global amino acids reduction and lipid remodeling, comprising phosphatidylcholines, sphingomyelins and lysophosphatidylcholines. The regression plots in Figures 4 and 5 represent the linear regression between scatter plot coordinates of the first principal component of the PCA or t1 (x-axis) and the relative concentrations, sums or ratios of metabolites (y-axis) of the most important polar metabolites and lipids found in both multivariate supervise models. Figure 4 suggests that spermine concentration increases with t1 (i.e. whilst the isoform-associated metabolic profile gets closer to the *Opa1^−/−^* profile and farthest from the *Opa1^+/+^* profile) and in *Opa1^−/−^* compared to *Opa1^+/+^* cells, while less protonated polyamines (putrescine and spermidine) are relatively decreased, while trans-hydroxylated proline (t4-OH-Pro) relative concentrations parallel that of amino acids concentrations.

Figure 5 reports the lipids’ data, showing a linear increased ratio of two sphingomyelins (SM18:0 and SM16:0) and hydroxylated sphingomyelins (SM:OH 22:1 and SM:OH 22:2) with t1. Conversely, the absolute concentration of polyunsaturated diacyl phosphatidylcholines (PUFA aa) as well as that of the ratio between polyunsaturated and monounsaturated (MUFA aa) diacyl phosphatidylcholines decrease with t1. For acyl-alkyl (ae) species, we also observed a decrease in the MUFA to SFA (saturated fatty acid) ratio with t1.

## Discussion

Our study provides compelling evidences for the high power of metabolomics to establishing and hierarchizing the pathogenicity of *OPA1* genetic variants expressed in an isogenic genetic background deleted for the endogenous *Opa1*. This strong predictability is essentially related to the statistical power of metabolomics, to the fact that all variants were expressed into the same nuclear background, rendering profiles highly comparable and to the growth condition in galactose that exacerbates mitochondrial dysfunctions. Indeed, as already reported, the absence of glucose and the presence of galactose restrict glycolysis rate and promote ATP production by the mitochondrial respiration (32). Under these conditions, cells exhibiting mitochondrial defects are slowly growing and more vulnerable to cell death (33).

PCA of the metabolome including MEF with *Opa1*^+/+^, *Opa1*^−/−^ genes and transfected with human *OPA1* isoforms (Fig. 1) stratified the metabolomic signatures of the *OPA1* variants according to their progressive order of pathogenicity. It should be stressed that PCA is an unsupervised method meaning that samples were grouped on the basis of their metabolic profile by the algorithm with no integration of the severity parameter when constructing latent variables (i.e. principal components). As expected from the PCA results, excellent predictive models were e further evidenced using the supervised OPLS(−DA) approaches, integrating PCA score of the first PC or t1 as a proxy for clinical severity (for the OPLS model). These supervised models enabled identifying the most discriminating metabolites with respect to the clinical severities.

Furthermore, the power of the metabolomics analysis allowed to precisely assess how much the different MEF cell lines differ from the ISO1control, containing the wild-type human cDNA, and from the *Opa1^−/−^* background, considered as the maximal pathogenic condition. Indeed, the p.R445H variant, responsible for the most severe syndromic phenotypes, turned out to be the closest to *Opa1^−/−^* genotype, behaving almost as a null allele. In contrast, the hypomorphic p.I382M variant, almost without pathological consequence by itself (22–24), plots very close to ISO1 in t1 but was not significantly different (*P*-value = 0.056, Wilcoxon test) in this latent variable. Through this discrete though no significant shift of I382M MEFs samples to the right of ISO samples (higher t1 scores) and toward dysfunctional variants metabolomics proves to be highly sensitive to the impact OPA1 function has on cell metabolic phenotype. This result found *in vitro* perfectly matches with clinical practice where I328M alone is not correlated with optic atrophy. It’s also reasonable to expect an overt metabolic distinction between I382M and ISO MEFs with an increased samples size. This finding is encouraging because, in a previous study, we were unable to identify any biochemical data distinguishing MEFs carrying this hypomorphic variant from ISO1 MEFs, since neither the mitochondrial network, nor the respiratory activity or the mtDNA were altered, in contrast with what we observed for the other pathogenic variants (31). Noteworthy, targeted metabolomics analysis of fibroblasts from a cohort of DOA patients failed to reveal any significant signature (34), while we did evidence a specific signature involving the purine metabolism in patients’ plasma, but without being able to discriminate the different *OPA1* mutations examined (35).

Thus, only the combined use of metabolomics and MEFs expressing *OPA1* alleles proves to be valid for highlighting metabolic changes discriminating genetic variants. From our results, this is expected to be truth even for the hypomorphic p.I382M allele, for which the pathogenicity remains questionable to date.

From these signatures classifying the variants according to their severity, we isolated the most discriminating metabolites responsible for generating this hierarchy, which consisted in the increase in spermine/spermidine ratio and the decrease of the sum of amino acids, t4-OH-Pro, SM OH 22:1, SM OH 22:2, SM 18:0/SM16:0 ratio, PC PUFA aa, PC PUFA/MUFA aa, PC MUFA/SFA ae ratios. Such a decrease in all amino acids was already observed in fibroblasts from patients with the Leber Hereditary Optic Neuropathy (LHON), the other major form of mitochondrial optic neuropathy, witnessing an ER stress with impairment of protein synthesis due to mitochondrial deficiency (34). This might be related to the fact that protein synthesis is one of the most energy-consuming cellular processes, or be a consequence of an alteration of the autophagic process (15). The decreased concentrations of hydroxyproline found here is in full agreement with this hypothesis since hydroxyproline levels reflect the turnover of collagen, which synthesis is one of the main fibroblast functions. The deregulation of polyamines (putrescine, spermine and spermidine) metabolism, also observed in the optic nerves of the *Opa1^+/−^* mouse model (36), in LHON fibroblasts (34) and in glaucoma patients (37), is known to be related with mitochondrial deficiency, and plays a crucial role in the post-translational processing of the eIF5A translation factor, by activating the protein translation activity (38). Therefore, the mitochondrial dysfunction related to *OPA1* mutations and the associated patho-mechanism could be related to a protein synthesis stress, through the alteration of polyamines metabolism and eIF5A maturation.

The other main alteration disclosed here concerns the phospholipid profiles. The increased concentrations of two hydroxylated sphingomyelins (SM OH 22:1, SM OH 22:2), as well as those of the SM18:0/SM16:0 ratio are highly predictive of variants severity. SMs play a crucial role in membrane structure and function, i.e. by forming lipid raft in association with cholesterol, by acting as second messengers themselves, by their enzymatic transformation in other highly informative molecules like ceramides, and by their contribution to myelin sheath composition. Little is known about cellular functions of the specific sphingomyelin species identified in this study. Nevertheless, SM18:0 specifically plays a role in protein export by acting as the natural ligand of p24 proteins (39). These p24 proteins facilitate cargo transport from ER to the Golgi apparatus, and under endoplasmic stress the increased SM18:0 concentration might contribute to ER relief, by enhancing cargo transfer to the Golgi apparatus. In our study, the levels of SM(OH) 22:1 and SM(OH)22:2 increased with the clinical severity of the *OPA1* mutations. Correlation between SM(OH)22:1 and SM(OH)22:2 concentrations observed here has been already described in a physiological context and was imputed to sphingolipid-specific desaturase activity (40). The biological meaning of this variation is unclear in the context of OPA1 dysfunction and to the best of our knowledge, the specific roles of these hydroxyl-sphingomyelin species have not been explored in any other pathology, so far. The increased SMs concentrations we observed is in apparent contradiction with what we have previously found in the *Opa1^+/−^* mouse optic nerve, where 10 sphingomyelins were decreased (36). These opposite results may reflect the differences between the tissues, with probably different pathways activation for sphingomyelin synthesis and catabolism in retinal ganglion cells and fibroblasts. Nevertheless, all our studies point to an alteration of sphingomyelin metabolism that needs to be further investigated as far as sphingomyelins play crucial roles in myelination and in the pathophysiology of mitochondrial optic neuropathies (41).

As for other different models of OPA1 dysfunction previously studied (34, 36, 42), we found that the phosphatidylcholines’ concentrations were also highly altered. The most discriminative features of variant severity were the decrease of the sum of polyunsaturated diacyl phosphatidylcholines (PUFA aa) and a decreased saturation of the diacyl phosphatidylcholines (PUFA/MUFA aa ratio) and alkyl species (MUFA/SFA ae). As phosphatidylcholines are the most important components of cell membranes, our observations suggest that membrane remodeling due to the alterations of mitochondrial dynamics and to the ER stress could affect the biosynthesis and transport of phospholipids between these two cellular compartments. Interestingly, decreased concentration of unsaturated phospholipids in cellular membranes was shown to trigger endoplasmic stress through non-classical pathways (43).

To conclude, the combination of the metabolomic approach with the analysis of *OPA1* mutated alleles expressed in the *Opa1* knock-out MEF model allowed to accurately classify and hierarchize *OPA1* variants according to their pathogenicity, clearly discriminating among the different missense variants. This strategy may further represent a relevant tool to predict the progression of the disease, to identify therapeutic targets and to assess readily their efficiency as surrogate biomarkers. Ultimately, this versatile approach could be useful for any rare disease related to metabolic perturbations.

## Materials and Methods

### Cells and culture conditions

MEFs with their native Opa1 endogenous gene (Opa1^+/+^), MEFs with deleted Opa1 gene (OPA1^−/−^) and Opa1^−/−^ MEFs transfected with the human wild-type OPA1 isoform 1 (the original transcript identified, RefSeq: NM_015560.2, ISO1), or human OPA1 isoform 1 carrying c.1146A > G (p.I382M), c.1316G > T (p.G439V), c.1334G > A (p.R455H) or c.1807G > C (p.D603H) pathogenic variants, previously described in (31, 44, 45) were used. The retroviral expression vector was co-transfected with the ecotropic retroviral packaging vector pCLEco into Hek 293 T cells. Retroviral stocks were harvested 48 h after transfection and used to infect MEF cultures. To achieve a stable cell pool, the selection with antibiotic puromycin lasted as long as the control (untransduced) cells completely die (7–10 days). The concentration of the antibiotic in culture was then reduced and, after 1 month, was removed entirely. The antibiotic selection with low concentration of puromycin was cyclically performed and the OPA1 expression in cells was regularly checked.

MEFs were grown in a high-glucose (25 mM) Dulbecco’s modified Eagle medium (DMEM, Life Technologies) supplemented with 10% fetal bovine serum (FBS, South America, Gibco, Life Technologies), 2 mM L-glutamine, 100 U/ml penicillin and 100 μg/ml streptomycin, in an incubator with a humidified atmosphere of 5% CO_2_ at 37°C. Twenty-four hours before harvesting, cells were grown in glucose-free DMEM (Life Technologies) supplemented with 5 mM galactose, 2 mM L-glutamine, 5 mM sodium pyruvate and 5% FBS (DMEM-galactose).

### Metabolites extraction

For each cell line, samples were collected in quintuplicate. When approximately 70% confluence was reached, the culture mediums were removed, and cells were washed twice in ice-cold phosphate-buffered saline (PBS) solution and harvested by scraping. The supernatants were discarded after centrifugation (3000 g for 5 min at 4°C) and the cell pellets were resuspended in 400 μl of ice-cold PBS solution, and centrifuged again in the same conditions. The supernatant was discarded, and the pellets immediately frozen by throwing in liquid nitrogen for 5 min and then stored at −80°C until analysis. Metabolites extraction was performed by adding 100 μl of ice-cold ethanol/PBS (85:15, v/v) solution to the cell pellets. After vortexing, the mixture was transferred to a 0.5 ml homogenizer tube prefilled with ceramic beads. Cell lysis were achieved in a Precellys®24 homogenizer (Bertin instruments, Montigny-le-Bretonneux) by two cycles of grinding (40 sec at 6500 rpm, followed by 30 sec at 6000 rpm) at 4°C. The resulting homogenates were centrifuged at 20000 g for 10 min at 4°C and the supernatants stored at −80°C until analysis.

### Metabolomics analysis

We applied a targeted, quantitative metabolomic approach to the cell extracts using the Biocrates technology (AbsoluteIDQ® p180 kit, Biocrates Life sciences) in an AB Sciex QTRAP 5500 (Life Sciences SCIEX) mass spectrometer. Samples were prepared according to the Biocrates Kit User Manual. Briefly, after thawing on ice, 10 μl of each sample (cell-lysate homogenate supernatants) were added to the center of the filter placed on the upper wall of the well in a 96-well plate. Metabolites were extracted in a methanol solution using ammonium acetate after drying the filter spot under nitrogen flow and derivatizing with phenylisothiocyanate. The extracts were finally diluted with mass spectrometry running solvent before mass spectrometry analysis. This kit enables quantification of up to 188 different endogenous molecules, including acyl-carnitines (40), amino acids (21), biogenic amines (21), glycerophospholipids (90), sphingolipids (15) and sugar (1). Flow-injection analysis (FIA-MS/MS) was used for quantifying acyl-carnitines, glycerophospholipids, sphingolipids and sugar, whereas liquid chromatography (LC) allowed the separation of amino acids and biogenic amines prior to detection with mass spectrometry (LC-MS/MS).

### Data cleaning and normalization

After validation of the three levels of QCs used in the kit and before statistical analysis, the raw data were examined to exclude metabolites with more than 20% of concentration values below the detection limit. Since the samples were analyzed in two different batches, to account for batch effect, the five *Opa1^+/+^* samples were analyzed in both batches and one of them was used to normalize all the samples in each batch (sample normalization). The effectiveness of sample normalization was then verified using PCA on the remaining eight WT samples. Metabolite concentrations in each sample were then normalized with respect to the total sum of metabolites in the given sample (row normalisation). Row normalisation avoids finding spurious between-samples differences merely due to differences related to the number of cells collected for each sample.

### Statistical analyses

Relative metabolite concentration between *Opa1^+/+^* and *Opa1^−/−^* were compared using non-parametric Mann-Whitney-Wilcoxon test.

Metabolites were scaled to have zero mean and unit variance scaling before submission to unsupervised and supervised algorithms. PCA was used as an unsupervised approach for outlier detection, based on Hotelling’s T2 distance, and identification of similar samples grouping together in the scatter plot. In the supervised analysis the X matrix of predictive variables was composed of metabolite concentrations. We applied two supervised analyses: orthogonal projection to latent structures (OPLS) alone and associated to discriminant analysis (OPLS-DA). In the OPLS-DA model, *Opa1^+/+^* and *Opa1^−/−^* MEFs were included in the Y vector as qualitative attributes. In the OPLS model sample coordinates of the five human transfected isoforms (i.e. ISO1, p.I382M, p.D603H, p.G439V and p.R445H) in the first principal component (PC1) was considered as the quantitative predicted variable (Y vector) whilst the X matrix was as before the metabolite concentrations. To avoid selecting optimistic over-fitted models, predictive capabilities of the final OPLS (DA) models were evaluated by cross-validation using cross-validated *R^2^Y* (*Q^2^Y*_cum_ or goodness of prediction), cross-validated analysis of variance (CV-ANOVA) test and the goodness of prediction of permuted models (*Q^2^Y*_cum-perm_). Models with a low degree of overfitting are characterized by *Q^2^Y*_cum_ > 0.5, negative *Q^2^Y*_cum-perm_ and are significantly more discriminant than the null model (CV-ANOVA *P*-value < 0.05). In OPLS-DA predictive model, selection of metabolites of interest was made through the combination of two pieces of information: VIP and the loading between the metabolite in the X matrix and the pLV(s) of the OPLS-DA model. Only metabolites with a VIP value larger than 1 and (absolute) high loading values were considered as important in the metabolomics signature. In the OPLS model, the volcano plot was formed by Spearman ρ correlation coefficients calculated between PC1 and each metabolite in the x-axis and VIPs in the y-axis. This way, we put together univariate and multivariate analysis in one plot, both capturing agreement between metabolite concentration and latent variables. More information about PCA- and PLS-based methods can be found in ‘Supplementary information: multivariate statistical analysis.’

## Supplementary Material

Supplementary_information_10-3-20_ddaa047

Supplementary_Table_S1_ddaa047
