# Supplementary material for: Metabolomics hallmarks OPA1 variants correlating with their in vitro phenotype and predicting clinical severity
Source: Hum Mol Genet. 2020 Mar 23;29(8):1319–29. doi: 10.1093/hmg/ddaa047 (PMC7254852; doi:10.1093/hmg/ddaa047)
Supplement: Supplementary_information_10-3-20_ddaa047 [file supplementary_information_10-3-20_ddaa047.doc]

**Supplementary information**

**Supplementary figures**

**
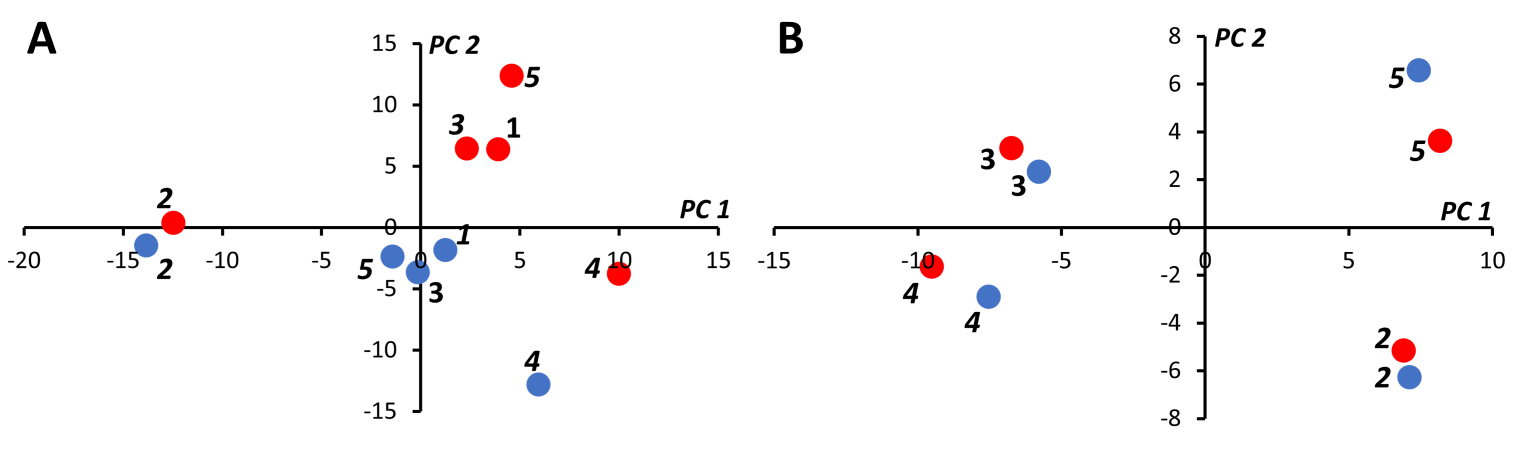
**

**Fig. S1. Validation of the results of sample normalisation by inspecting the first principal plan of the PCA.** Five *Opa1^+/+^* MEFs samples were analysed in batch 1 (blue circles) and batch 2 (red circles). **A**- In the absence of sample normalisation, the same samples do not group together, save for sample 2, a batch effect can also be detected and captured by the second principal component (PC2) with samples analysed in batch 1 having negative PC2 coordinates and almost all samples analysed in batch 2 having positive PC1 coordinates. **B**- When samples were normalized using sample 1, batch effects are no longer detected in the first principal plan and the same samples group together independently of the batch analysed.

**Supplementary materials and methods**

**Multivariate statistical analysis**

In this section only PCA and PLS related methods: (O)PLS-DA methods will be introduced. PCA and PLS methods belong to the family of projection methods. Indeed, PCA and PLS related methods project data from their original high dimensional space (e.g., **X** matrix of measured metabolites) to a reduced space formed by the so-called latent variables (LVs). These LVs are linear combinations of the measured metabolites (or overt variables, columns of **X**) and they are considered to be a much better approach to the phenomenon under study (i.e., OPA1 mutation effect on metabolome) but they cannot be directly measured. Projection methods are very frequently used in omics literature may be because data matrices in these approaches are “short and wide” (i.e., many more variables than samples) hindering the use of “classical” multivariate methods like multiple linear and logistic regressions.

Principal component analysis. In PCA latent variables are called principal components (PC) and they are constructed iteratively with the first PC (PC1) spanning the direction that captures most of the information (or covariance in this case) contained in the covariance matrix (i.e., when samples are projected in this direction the variance is maximum compared to any other direction formed by a linear combination of the column of **X**, i.e., metabolites). Then the second principal component is built in a direction orthogonal to PC1 and capturing the maximum of information in this space. The process is repeated until an “enough” amount of covariance is captured by PCs (generally between 60 and 70%). This is generally achieved with 4 to 5 PCs but depends on the structure of data matrix. The maximum number of PCs is fixed by the rank of the matrix (i.e., in metabolomics this is at most the number of samples in metabolomics). In PCA the matrix **X** is approached as the multiplication of two matrix **T** and **P**^T^ (**X** ≈ **TP**^T^) where **T** contains the scores (what we call here principal component), that’s the coordinates of the samples when projected to the low dimensional space formed by the LVs (i.e., PCs) and P contains the loadings, that is the coefficients of the linear combination of each variable to from the PCs. PCA is considered as an unsupervised method because only the information contained in the data matrix is used, regardless of any predicted or response variable.

Partial least square related methods. PLS methods, on the opposite, built latent variables in a similar way PCA does but the goal here is to maximize the correlation between the latent variable and the response variable (a vector in our case, called Y). When the second LV is orthogonal to the first (predictive) LV, the method is called OPLS. OPLS is more suitable for model interpretation as far as important metabolites are only those “important” in the predictive LV but this is not the case when more than one LV built from data matrix correlate with the response variable. When the Y is qualitative, the method is associated to discriminant analysis (DA) for between-group discrimination and is then called (O)PLS-DA.

One problem with supervised methods is overfitting meaning the model works very good in estimating the Y vector in the data set used for building it but has poor predictive capabilities when the same model is applied to another data set issue from the same population. This can be simply explained by the fact that in each data set natural fluctuations of the sampling process can randomly make a group of variable important ( a very similar situation occurs when performing multiple univariate tests, making necessary to correct risk I error inflation) leading to group separation (in the case of OPLS-DA, for example). To estimate the overfitting degree different diagnostic tests can be used based on cross-validation (CV) strategy. For CV data are randomly broken into *n* parts of approximately equal size and then a model is built with (*n*-1)/*n* parts and tested in the part left out. This process is repeated n times, leaving each time one part out for predicting the outcome. This way the prediction error sum of squares (PRESS) can be calculated as the squared differences between observed and predicted values for the data left out for prediction. Based on PRESS values, the fraction of the total variation in the Y vector that can be predicted by a latent variable called Q^2^Y can be calculated as Q^2^Y= (1- (PRESS/SS)) where SS, the sum of squares, is representative of the total variance in the Y vector. This way cumulative Q^2^Y (Q^2^Yc or Q^2^Y_cum_) is an estimation of the fraction of the variance of the Y vector the model can predict by the PLS model. Generally, Q^2^Yc >0.5 is regarded as good and Q^2^Yc>0.9 as excellent. Another way to test the performance of a PLS based method is cross-validated analysis of variance (CV-ANOVA). This test is based on the hypothesis that residuals (the part of Y vector non explained by the model) should be significant reduced in a predictive model compared to the residuals of a “random” (non- predictive) model. For predictive model the null hypothesis of equality of PLS model and random model should be rejected (P-value <0.05). Finally, if the entries of the Y vector are permuted while preserving the X matrix, the predictive capabilities of the PLS model should decrease, this fact supports the specificity of the correlation between the X matrix and the initial Y vector. It has been shown that predictive models have negative Q^2^cY or Q^2^Y _cum-perm_ when the correlation between the permuted and the original Y vectors is 0.

Once a PLS-based model is retained (Q^2^Y_cum_ >0.5, P-value (CV-ANOVA) <0.05, Q^2^Y _cum-perm_ <0) one is interested by the most important variable responsible for prediction of Y vector. Loadings are the coefficients in the linear combination of metabolites that forms the latent variables. This ways, higher loading values (negative or positive) indicate higher importance of the variable in a given latent variable. Variable importance for the projection or VIP summarizes the importance of each variable (metabolite) in all the latent variables of the model. It can be shown the variables with VIP value ≥ 1 are the most relevant for explaining Y. When combining loadings and VIPs for all variables in a plot it is easy to see what variables (metabolites) are important for the explanation of Y vector based on the data matrix X. “Natural” relation between VIP and loadings makes the plot to take a “V” shape and that is why its called volcano plot.

More detailed information about PCA and PLS-based methods can be found by the interested reader the book “Multi- and megavariate data analysis. Part 1: Basic principles and applications” from L. Eriksson.

**Table S1: Measured metabolites (μmol/L)**. Excel format.
